# Targeted use of automated estrus detection and strategies to improve fertility outcomes in lactating dairy cows

**DOI:** 10.1590/1984-3143-AR2026-0086

**Published:** 2026-09-21

**Authors:** Rafael Sisconeto Bisinotto, Phillip Martins Gondim Peixoto, Victoria Alice Bastos Primo, Maria Belen Ugarte Marin, Deniquer Klein Roese, Pedro Leopoldo Jerônimo Monteiro, Ricardo Carbonari Chebel

**Affiliations:** 1 Department of Large Animal Clinical Sciences, University of Florida, Gainesville, FL, USA; 2 College of Veterinary Medicine at Orange Park, Lincoln Memorial University, Orange Park, FL, USA; 3 Department of Animal Sciences, University of Florida, Gainesville, FL, USA

**Keywords:** fertility, pregnancy diagnosis, wearable devices, genomic testing

## Abstract

Reproductive performance of lactating dairy cows has improved largely over the past two decades. Such progress reflects advances in reproductive management practices, but also incorporation of fertility traits into genetic selection programs and improvements in cow comfort and health that have allowed high-producing dairy cows to better express their reproductive potential. This review summarizes recent developments in reproductive management strategies that have contributed to continued improvements in reproductive efficiency in lactating dairy cows. Increased availability of genomic data and automated monitoring technologies has created unique opportunities for the development of targeted management strategies tailored to specific cohorts of cows, which can reduce reproductive costs and allow for reorganization of routine tasks and labor utilization while maintaining reproductive performance. As the genetic makeup of high-producing dairy cows continues to evolve, understanding of the biological processes that control fertility will remain paramount so that novel technologies can be employed effectively as they become applicable to commercial farms. Strategies to improve reproductive performance of cows with inferior genetic merit for fertility and during the hot months of the year, among solutions to other physiological and environmental challenges, are expected to complement automated monitoring systems. Novel strategies for early identification and reinsemination of nonpregnant cows have allowed for the development of efficient resynchronization protocols.

## Introduction

Progressive dairy farmers, dairy scientists, and the allied industry have demonstrated a strong capacity for innovation and implementation of transformative technologies over the last decades. The development and refinement of hormonal protocols for synchronization of ovulation and timed artificial insemination (**AI**) allowed for greater control of breeding in high-producing dairy cows, which coincided with a drastic reversal of the longstanding trend of decreased fertility phenotypes in dairy cows starting in the early 2000’s (Stevenson and Britt, 2017). Besides its importance as a tool to improve reproductive performance, timed AI programs became a focal point in the transition from a traditional mindset to proactive and systematic reproductive management. More recently, widespread adoption of automated monitoring devices (**AMD**) was made possible by the scalability of hardware and data pipelines to meet the demands of large dairies. A variety of AMD for characterization of physical activity patterns based on step counts or head movement, oftentimes combined with monitoring of rumination time yield high accuracy for estrus detection in lactating dairy cows (Mayo et al., 2019).

Improvements in reproductive performance over the past two decades have reflected not only advances in reproductive strategies, but also broader advancements in genetics, health, and cow comfort that created conditions for these strategies to be effective. Genomic testing accelerated the genetic progress of dairy breeds and facilitated selection for milk yield concurrently with health, fertility, and feed efficiency (Ma et al., 2019). Inclusion of daughter pregnancy rate (**DPR**) and cow conception rate (**CCR**) in the calculation of lifetime net merit helped halt and eventually reverse the progressive decline in genetic potential for reproductive performance (García-Ruiz et al., 2016; Ma et al., 2019). Finally, advancements in heat abatement systems and cow comfort have allowed high-producing dairy cows to express their reproductive potential (Bewley et al., 2017; Ouellet et al., 2020).

The objective of this review is to discuss recent developments in reproductive management of lactating dairy cows. Particular attention is given to targeted reproductive management strategies based on automated estrus detection, use of GnRH to improve pregnancy per AI (**P/AI**) at reinsemination, and early detection of nonpregnant cows (Figure 1). Genetic selection for fertility traits and heat abatement are also considered briefly, not as primary areas of focus, but because they interact with the reproductive strategies discussed here. This review emphasizes research conducted in North American dairies, particularly studies focused on the use of AMD for targeted management of reproduction. Nevertheless, the biological concepts that underlie these strategies are broadly applicable to other dairy production systems. Protocols for pharmacological control of luteal function and ovulation developed elsewhere were considered to widen the scope of this review. However, we would like to frame these protocols as alternative tools for management of AI that can be used in various scenarios depending on availability and cost, rather than focusing on direct comparisons between synchronization programs.

**Figure 1:**
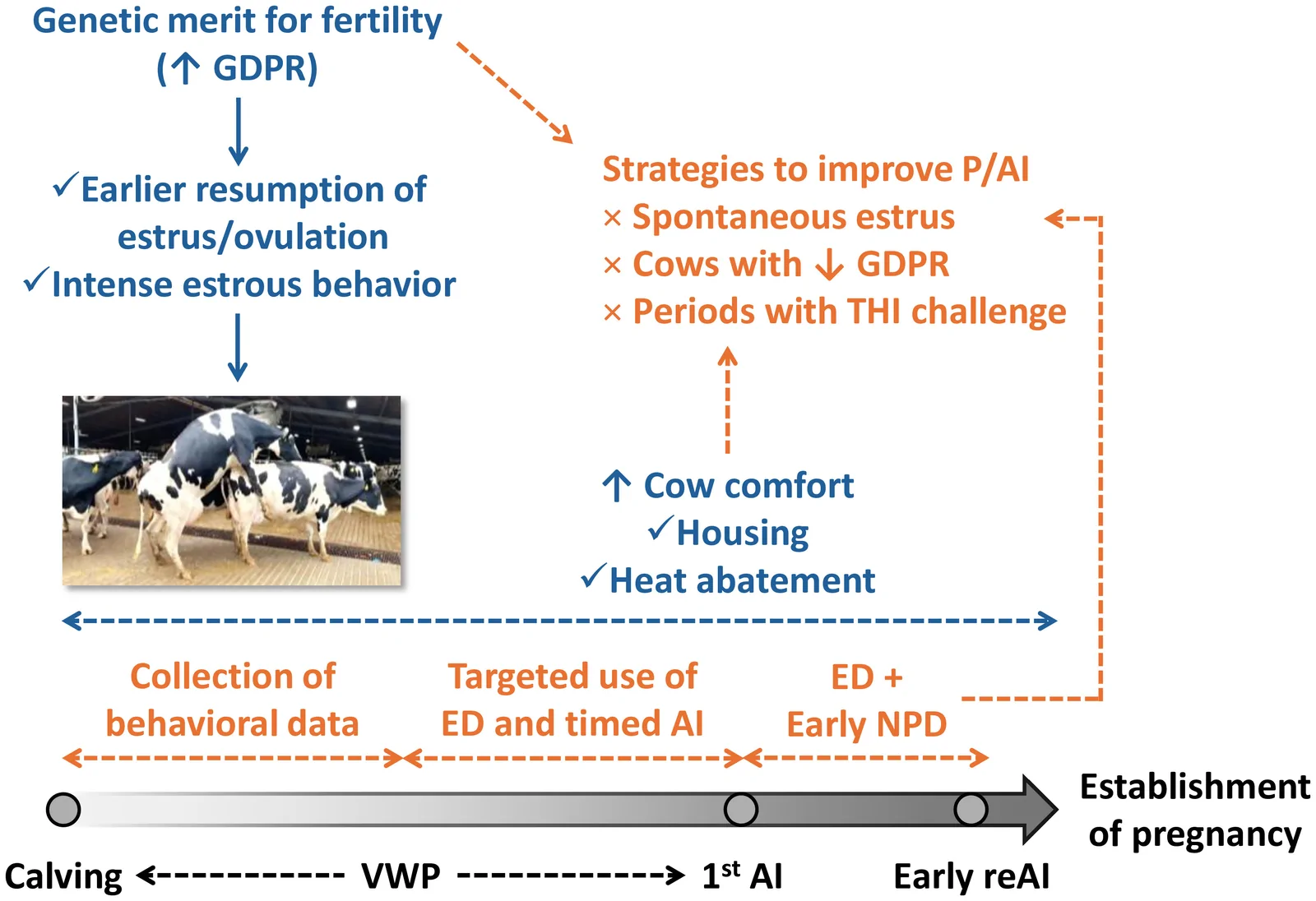
Diagram of reproductive strategies focused on targeted use of estrus detection (ED) and timed artificial insemination (AI) for first service postpartum and early reinsemination (reAI). Data on estrous behavior, health, and performance gathered during the voluntary wait period (VWP) is used to identify cows that become pregnant efficiently using ED (streamlining the flow of cow handling and labor allocation), whereas remaining cows are subjected to timed AI. Despite genetic selection for fertility traits and improvement in housing, strategies that increase pregnancy per AI (P/AI) in cows with low genetic daughter pregnancy rate (GDPR) and during periods with elevated temperature and humidity index (THI) benefit reproductive performance. Novel methods for early nonpregnancy diagnosis (NPD) are expected to reduce reAI intervals and increase pregnancy rate.

## Genetic selection for fertility and reproductive management

Genetic selection for fertility in the US has relied heavily on DPR. Daughter pregnancy rate is calculated as a linear transformation of time to pregnancy into a 21-day pregnancy rate basis (Kuhn et al., 2004; VanRaden et al., 2004). Cows with high estimated breeding values (**EBV**) for DPR have greater pregnancy rate and fewer days open compared with counterparts with low DPR (Melo et al., 2025). Yet, there are multiple biological processes that can explain why cows with high DPR become pregnant faster. For instance, pregnancy rate can be improved by selecting for 1) cows capable of resuming ovulation earlier postpartum (Galvão et al., 2010), 2) cows with greater uterine capacity that become pregnant with fewer services (Moraes et al., 2018), 3) cows with intense sexual behavior that are more likely to be inseminated under management systems that rely on estrus detection (Chebel et al., 2025), or 4) any combination of these traits. Understanding the mechanisms that have been impacted by genetic selection for cow fertility is expected to provide information that can be applied to streamline management practices and further improve reproductive performance.

Researchers from Ireland developed a model based on intentional selection of females with high or low fertility based on EBV for calving interval (Cummins et al., 2012a; Butler, 2013), which is analogous to DPR as it is also a function of time to pregnancy. This model resulted in two cohorts with diverging fertility phenotypes. As expected, high fertility cows had greater pregnancy rate and fewer days to pregnancy compared with low fertility herdmates (Cummins et al., 2012a). High fertility cows had larger corpus luteum (**CL**) and greater concentrations of progesterone during diestrus (Cummins et al., 2012b). Diameter of the ovulatory follicle and estradiol concentrations during proestrus and estrus did not differ between the two cohorts. However, incidence of ovulation without manifestation of estrous behavior (i.e., silent ovulation) and of estrous behavior not followed by ovulation (i.e., ovulation failure) were greater in low compared with high fertility cows (Cummins et al., 2012b). These results suggest that the difference in reproductive performance between low and high fertility cows (hypothetically, also between cows with low and high DPR) might be mediated by reduced hypothalamic sensitivity to estradiol positive feedback, deficiency in estradiol-induced LH release, or diminished ovarian response to LH. Using a similar model, researchers at the University of Minnesota created two contemporaneous cohorts of Holstein cows: 1) unselected Holsteins were generated by continuous use of top bulls for milk yield commercially available in the early 1960s; 2) contemporary Holsteins were generated using the same genetic practices available to US dairy producers over the years. Besides expected differences in milk production, this model captured a decline in EBV for DPR from 20.3 to 0.4% observed for Holstein females between 1958 and 2003 (Council on Dairy Cattle Breeding; https://webconnect.uscdcb.com/#/summary-stats/genetic-trend, accessed 06/27/2026). Field studies depicted a delay in resumption of ovulation postpartum observed in contemporary cows compared with unselected counterparts (Weber et al., 2003). Recent studies have confirmed the relationship between DPR and resumption of reproductive function postpartum. Genomic DPR (**GDPR**) was linearly associated with the proportion of cows detected in estrus within the first two months postpartum and the proportion of cows that resumed ovulation by 49 days in milk (**DIM**; Chebel and Veronese, 2020; Peixoto et al., 2023; Peixoto et al., 2024; Chebel et al., 2025). Genomic DPR was also associated with intensity of estrous behavior. Chebel and Veronese (2020) observed a negative association between GDPR and rumination nadir during estrus and a positive relationship between GDPR and the likelihood of high-intensity estrus alerts.

## Resumption of estrous behavior and ovulation postpartum

Early resumption of estrous behavior and ovulation postpartum continues to be a critical first step towards achieving reproductive efficiency in lactating dairy herds. Cows that resumed ovulation by 50 to 60 DIM had greater insemination hazard, greater P/AI to first service following estrus detection or timed AI, and greater hazard of pregnancy (Gümen et al., 2003; Santos et al., 2009; Galvão et al., 2010). It is interesting to note that, even among cows that resume ovulation within the first two months of lactation, earlier resumption of ovulation was associated with greater reproductive performance (Galvão et al., 2010). A similar relationship between detection of estrous behavior postpartum and reproductive performance has been observed. Using a collar-mounted AMD that characterizes changes in overall physical activity and rumination based on head movements, several research groups concluded that cows detected in estrus before the end of the voluntary wait period (**VWP**) have greater hazards of insemination and of pregnancy (Borchardt et al., 2021; Rial et al., 2022; Chebel et al., 2025). The intensity of behavior measured by AMD during the VWP has also been associated with subsequent reproductive performance. For instance, rumination nadir was negatively associated with the probability of pregnancy after the first service postpartum in a study that included cows that received AI following estrus detection, timed AI, embryo transfer (**ET**) after estrus detection, or timed ET (Chebel et al., 2025).

Anestrus and anovulation have been often used interchangeably to define cows that fail to resume cyclic ovarian function and sexual behavior after calving. Nevertheless, evolution of technologies that allow for identification of CL and characterization of estrous behavior in large populations of cows has highlighted the need for adherence to specific terminology. Throughout this review, anovulation will be used in reference to cows that do not resume ovulation within a certain interval postpartum. Conversely, anestrus will be used in reference to cows that do not display behavioral estrus within a certain interval postpartum. The distinction between anovular and anestrus cows is relevant because current strategies for automated estrus detection during the VWP have only moderate predictive value to identify cows that resumed ovulation. Thus, a better understanding of the physiological conditions of cows monitored using AMD is needed to improve the efficacy of target reproductive strategies based on estrous behavior.

In a meta-analysis encompassing studies in which cows were monitored for estrous behavior during the VWP, the average sensitivity and specificity of estrus alerts to identify cows that resumed ovulation were 70 and 60%, respectively (Borchardt et al., 2025). In fact, 12% of the cows detected in estrus had not resumed ovulation and 65% of cows classified as being in anestrus by AMD had resumed ovulation based on progesterone concentrations (Borchardt et al., 2025). The use of AMD for identification of cows that resumed ovulation postpartum based on detection of CL ≥ 20 mm in diameter by transrectal ultrasonography yielded similar results (Peixoto et al., 2023; Peixoto et al., 2024). Overall sensitivity and specificity of estrus alerts between 8 and 46 DIM to identify cows with CL were 59 and 61%, respectively. False discovery rate (proportion of anovular cows with estrus alerts) was 22% and false omission rate (proportion of anestrus cows with CL) was 61%. Marginal predictive values for AMD to identify cows that resumed ovulation postpartum are related to animal- and technology-related factors. From a biological perspective, cows with low GDPR have less intense estrous behavior that can be missed by AMD and an increased incidence of ovulation events not associated with estrous behavior (Cummins et al., 2012b; Chebel and Veronese, 2020). Milk production has also been negatively correlated to the estrus duration, frequency of mounting activity, and activity peak characterized using AMD (Lopez et al., 2004; Marques et al., 2020). Moreover, AMD technology was developed to identify cows in estrus during breeding periods (i.e., later in lactation past the VWP). Estrous behavior is weaker early postpartum and increases in intensity as DIM progresses (Marques et al., 2020), potentially increasing the incidence of false negatives for AMD identification of cows that resume ovulation after calving. Mayo et al. (2019) compared the accuracy of various AMD to identify periods of estrus defined by circulating concentrations of progesterone in lactating cows ranging between 45 and 85 DIM. False omission rate ranged from 42.9 to 80.7% across different brands. It is interesting to note that visual observation of estrous behavior did not fare better, with false omission rates of 68.1% for observation of standing behavior and 74.1% for a more comprehensive behavioral score.

As discussed in the following section, reproductive strategies have been developed to maximize the use of estrus detection by targeting cows with high likelihood of being detected and becoming pregnant. Therefore, increasing the precision of AMD to characterize reproductive function during the VWP is paramount. Relocation of cows to a different group changes activity and rumination patterns and, therefore, has been hypothesized to increase the likelihood of false estrus alerts (Bruinjé et al., 2023; Laplacette et al., 2024). Peixoto et al. (2024) assessed the potential impact of moving cows between pens on the accuracy of automated estrus alerts to identify cows that resumed ovulation postpartum. However, censoring estrus alerts for cows moved between pens from 0 to 72 hours relative to the alert decreased overall accuracy. These results underscore the need for novel approaches to the use of AMD data as a proxy for characterization of ovarian function early postpartum.

## Targeted reproductive management

Targeted reproductive strategies are based on the premise that data available before the first service postpartum can be used to capture the physiological potential of individual cows and determine the optimal reproductive strategy. Rial et al. (2022) compared a targeted reproductive approach for first AI postpartum against two blanket strategies, in which 1) all cows received timed AI program using the Double-Ovsynch protocol, or 2) all cows were subjected to estrus detection for 24 ± 3 days and those not inseminated received timed AI using the Ovsynch protocol with progesterone supplementation. Cows assigned to the targeted reproductive management were monitored for estrous behavior using AMD from 21 to 49 DIM – cows with at least one estrus alert were allowed an estrus detection period of 31 ± 3 days after the VWP, whereas cows not detected in estrus during the VWP were exposed to estrus detection only for 17 ± 3 days. The targeted reproductive management resulted in greater hazard of pregnancy and a similar proportion of pregnant cows by 150 DIM compared with the blanket use of the Double-Ovsynch for first service. However, no major benefit was observed for the targeted reproductive management compared with the use of a fixed estrus detection period for all cows independent of estrus alerts during the VWP. Similar results have been reported by the same research group using slight variations of this design (Laplacette et al., 2024).

Gonzalez et al. (2023) compared the performance of blanket use of the Double-Ovsynch protocol (control reproductive program) with a targeted reproductive management approach that classified cows according to automated estrus alerts during the VWP. Different from Rial et al. (2022), intensity of estrous behavior measured as heat index (**HI**) was considered for allocation of cows to reproductive protocols. Cows assigned to the targeted management that were not detected in estrus during the VWP or that had only low-intensity estrus alerts (HI < 70) were both subjected to the Double-Ovsynch protocol and received timed AI similar to the control program. On the other hand, cows with high-intensity estrus alerts (HI ≥ 70) were monitored using AMD for 42 days after the VWP. After this period, cows that were not inseminated in estrus were subjected to timed AI using the Double-Ovsynch protocol. Overall, 52.5% of cows in the targeted reproductive management (n = 731) displayed high-intensity estrous behavior during the VWP and were exposed to estrus detection. Of those exposed to estrus detection, only 4.0% of primiparous and 9.4% of multiparous cows were not inseminated following an AMD alert and received timed AI for first service. Therefore, 55.8 and 42.9% of all primiparous and multiparous cows initially enrolled in the targeted reproductive management were inseminated in estrus, respectively, without the need for hormonal treatments. One key result from this experiment is that the selection criteria allowed for a considerable portion of the multiparous to be inseminated in estrus, while maintaining the overall P/AI (44.7%; n = 468) similar to that observed in control cows subjected to the Double-Ovsynch protocol (41.0%; n = 463). On the other hand, the same criteria applied to primiparous cows reduced overall P/AI (27.4%; n = 259) compared with the control reproductive program (37.6%; n = 277).

These results highlight the importance of the classification strategy used to determine the optimal reproductive strategy for specific cohorts. For instance, the criteria of HI ≥ 70 used by Gonzalez et al. (2023) was defined based on hazard of pregnancy throughout lactation. It is possible that different criteria based on expected results for the first AI postpartum can improve P/AI in cows subjected to targeted reproductive management. Rial and Giordano (2024) classified cows based on automated estrus alerts during the VWP (from 21 to 49 DIM) and risk factors for poor reproductive performance (highest tercile for milk yield, lowest tercile for GDPR, occurrence of at least one case of health disorder). Estrus alert during the VWP had the greatest impact on the hazard of insemination after the end of the VWP, whereas absence of risk factors for poor reproductive performance had the greatest impact on P/AI to first service (Rial and Giordano, 2024). As a result, the two classification variables had an additive effect on pregnancy hazard and proportion of pregnant cows by 150 DIM. Despite ongoing efforts to refine selection criteria, targeted reproductive strategies have been shown to allow for selective interventions that reduce the use of hormonal treatments for synchronization of ovulation, creating an opportunity for dairy producers to minimize animal handling and reorganize labor utilization.

It is important to highlight that the use of Double-Ovsynch as the blanket timed AI program in control reproductive management and as failsafe for cows not inseminated during periods of estrus detection in these experiments reflects the popularity of this protocol amongst US dairies. Synchronization programs that incorporate estradiol cypionate (EC) and progesterone-releasing devices in addition to GnRH and PGF_2α_ are potential alternatives to Double-Ovsynch in other countries where the use of estradiol esters is allowed. For instance, Consentini et al. (2025) compared four programs for management of the first AI postpartum including the Double-Ovsynch (Ovs+Ovs). Alternate protocols included 1) Ovs+OvsP4/E2 wherein cows received a progesterone insert and the final GnRH injection was replaced by EC, reducing animal handling and labor needed for an afternoon injection; 2) PreP4/E2+Ovs wherein the first Ovsynch was replaced by a progesterone insert and concurrent injections of PGF_2α_ and EC, also reducing animal handling compared with Double-Ovsynch; and 3) PreP4/E2+OvsP4/E2 which combined the managerial benefits of both alternate protocols. Both presynchronization strategies resulted in a large proportion of cows with CL at the start of the timed AI protocol (Ovs = 95.5 vs. PreP4/E2 = 90.8%; *P* = 0.04), ovulation after the first GnRH (Ovs = 64.3 vs. PreP4/E2 = 72.0%; *P* = 0.04), and presence of CL at the first PGF_2α_ (Ovs = 99.0 vs. PreP4/E2 = 98.0%; *P* = 0.54). Both timed AI strategies resulted in a large proportion of cows with synchronized ovulation at the time of AI (Ovs = 96.1 vs. OvsP4/E2 = 92.2%; *P* = 0.02). More importantly, P/AI on day 32 after AI did not differ among reproductive programs (Ovs+Ovs = 46.0%; Ovs+OvsP4/E2 = 45.5%; PreP4/E2+Ovs = 51.7%; PreP4/E2+OvsP4/E2 = 48.7%). These protocols have been deemed effective as blanket strategies for management of the first AI postpartum. It is reasonable to speculate that protocols involving EC and progesterone inserts can be used in targeted reproductive management strategies, and that the protocol choice will be influenced by product availability and cost instead of fertility considerations.

## Improving p/ai in cows reinseminated following estrus detection

The combined use of estrus detection and timed AI arguably provides dairy producers with the best strategy to maximize reproductive efficiency and herd profitability (Giordano et al., 2012; Galvão et al., 2013). Improving estrus detection efficiency increases insemination rate and reduces the interval between AI. However, the benefits of estrus detection rely heavily on the accuracy of estrus detection methods, which is intrinsically related to the P/AI in cows inseminated upon detected estrus (Giordano et al., 2012). Considering the widespread use of AMD in dairy herds, strategies to improve P/AI in cows inseminated following automated estrus detection will continue to gain importance. Such strategies focus on three major factors that influence the establishment of pregnancy in cows monitored using AMD: physiological conditions during preovulatory follicle development, ovulation following automated estrus alerts, and synchrony between AI and ovulation.

Studies from the 1970s have demonstrated that elevated progesterone concentrations during growth of the preovulatory follicle are linked to greater probability of pregnancy. For cows inseminated after spontaneous estrus, progesterone concentrations during the preceding diestrus were greater in cows that became pregnant compared with counterparts that failed to become pregnant (Folman et al., 1973). The beneficial effect of progesterone during development of the ovulatory follicle has been linked to reduced LH pulse frequency and appropriate progression of oocyte maturation (Santos et al., 2016). In addition to the impact of circulating concentrations of progesterone, studies have shown that reducing the interval from emergence of the ovulatory follicle to estrus and ovulation improves embryo quality and increases P/AI (Bleach et al., 2004; Cerri et al., 2009). Both concepts have been applied successfully to timed AI protocols (Santos et al., 2010; Bisinotto et al., 2013), but less emphasis has been placed on estrus detection programs. Treatment with GnRH on day 7 of the estrous cycle is highly efficient in inducing ovulation of the first wave dominant follicle (Vasconcelos et al., 1999). Besides forming of an accessory CL and increasing circulating progesterone concentrations, ovulation of the first wave dominant follicle is expected to increase the proportion of cows with 3-wave cycles and shorten the interval from emergence to ovulation (Ginther et al., 1989; Diaz et al., 1998; Cunha et al., 2021). Based on this rationale, our group tested the hypothesis that treatment with GnRH 7 days after AI delays return to estrus and increases P/AI in cows reinseminated following an AMD estrus alert (Gonzalez et al., 2024). Treatment with 172 µg of gonadorelin acetate induced ovulation in 79.1% of cows (110/139) and delayed return to estrus in cows previously inseminated following estrus detection (GnRH = 24.1 vs. Control = 22.5 days; AHR = 0.76, 95% CI = 0.60 to 0.97). Interestingly, treatment with GnRH did not delay estrus in cows that previously received timed AI (GnRH = 24.7 vs. Control = 24.9 days; AHR = 0.99, 95% CI = 0.83 to 1.20). The effects of GnRH on intensity of estrous behavior for cows reinseminated in estrus were modest. Treatment with GnRH reduced rumination nadir during estrus in primiparous cows (GnRH = -49.1 vs. Control = -41.6%; *P* = 0.03), but not in multiparous cows (GnRH = -43.3 vs. Control = -42.1%; *P* = 0.47). Treatment did not affect duration of estrus (GnRH = 16.5 vs. Control = 16.3 hours; *P* = 0.52) or the proportion of cows with activity peak greater than 90% (GnRH = 84.4 vs. Control = 81.9%; *P* = 0.38). Nevertheless, treatment with GnRH 7 days after a previous AI tended to increase (*P* = 0.06) P/AI in cows reinseminated in estrus (GnRH = 46.1, 149/323 vs. Control = 38.8%, 124/320).

Treatment of cows with GnRH at the time of AI has the potential to increase P/AI in cows at greater risk of ovulation failure and asynchrony between ovulation and AI. However, development of a targeted approach relies on understanding which are the cohorts of cows that benefit from GnRH treatment. Previous studies suggested that cows with low-intensity estrus alerts characterized by AMD are less likely to ovulate spontaneously and have more disperse ovulations compared with cows with intense estrus alerts (Burnett et al., 2018; Cerri et al., 2021). In cows monitored using AMD, treatment with GnRH at the time of AI increased P/AI in cows with low-intensity estrus alerts, but not in cows with high-intensity alerts (Burnett et al., 2022). Interestingly, GnRH treatment increased the proportion of cows with high-intensity estrus that ovulated within 48 hours from the alert, whereas no effect was observed in cows with low-intensity alerts. Primo et al. (2026) also evaluated the impact of GnRH treatment at the time of AI in cows monitored using AMD. Differently from Burnett et al. (2022), cows were categorized into low-intensity (HI ≤ 80), moderate-intensity (HI = 84 to 92), and high-intensity estrus alerts (HI ≥ 96) based on the distribution of estrous intensity. Treatment of cows detected in estrus using AMD with GnRH at the time of AI increased P/AI both in cows with low- and high-intensity estrus alerts, whereas no effect was observed in cows with estrus alerts of moderate intensity (Figure 2A).

**Figure 2:**
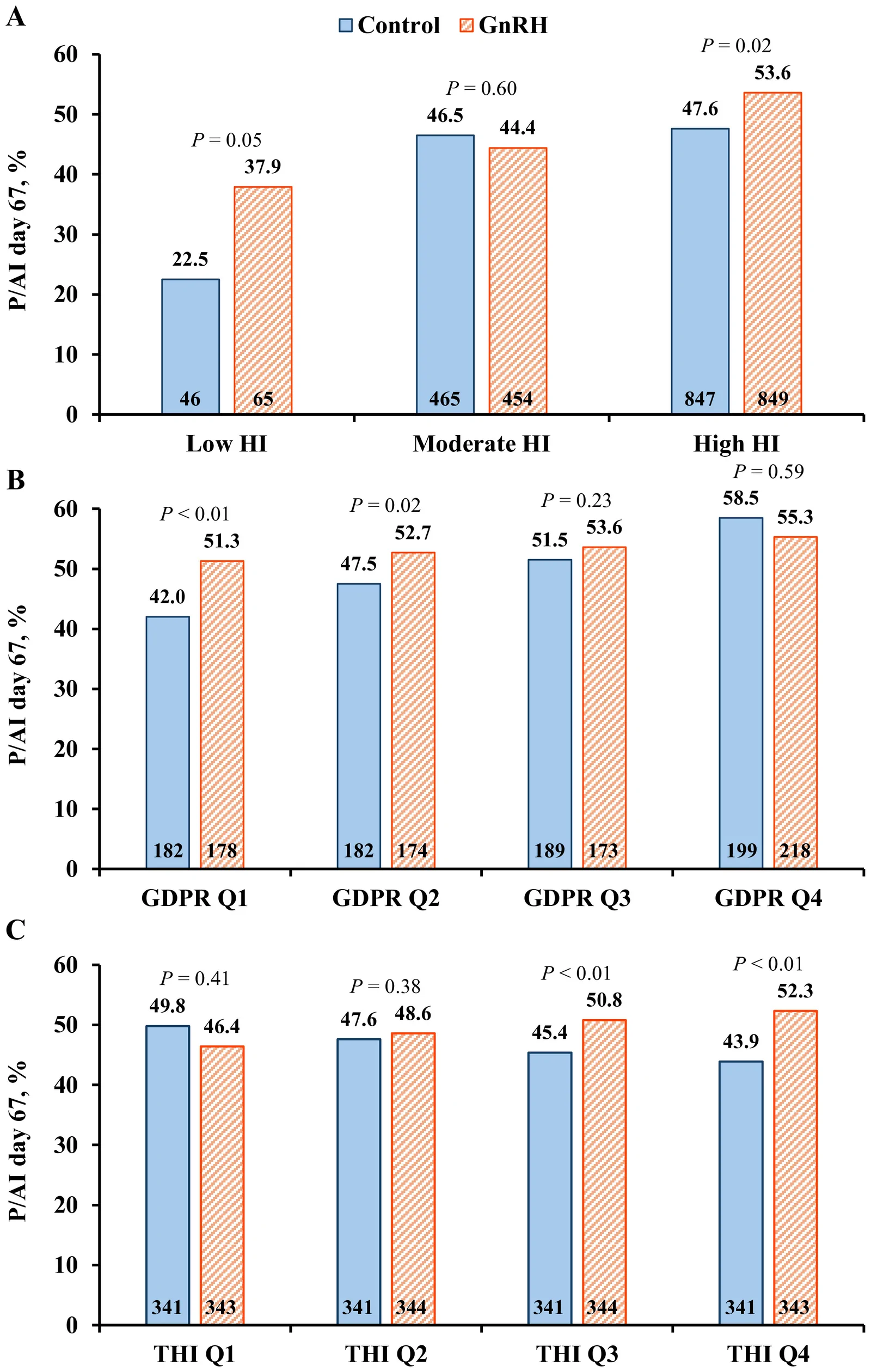
Pregnancy per AI (P/AI) for cows detected in estrus using automated monitoring devices (AMD) and treated with GnRH at the time of AI (orange bars) or untreated controls (blue bars). Panel A: estrus alerts categorized as low (HI ≤ 80), moderate (HI 84 to 92), or high heat index (HI ≥ 96). Panel B: cows categorized into quartiles of genomic daughter pregnancy rate (GDPR; quartile means: Q1 = -1.75, Q2 = -0.57, Q3 = 0.29, Q4 = 1.80). Panel C: estrus alerts categorized into quartiles of average temperature and humidity index (THI) in the 15 days preceding AI (quartile means: Q1 = 53.3; Q2 = 62.6; Q3 = 72.1; Q4 = 78.4). Numbers inside bars represent the number of AI events available for statistical analyses (Primo et al., 2026).

The results from Primo et al. (2026) also indicate that treatment with GnRH at the time of AI in cows monitored using AMD benefited fertility of those with low genetic merit for fertility and during periods when cows are under greater risk of heat stress. Based on the model developed in Ireland, cows with low GDPR are expected to have a greater incidence of ovulation failure compared with counterparts with high GDPR (Cummins et al., 2012b). Accordingly, treatment with GnRH at the time of AI increased P/AI in the bottom two GDPR quartiles, whereas no treatment effect was observed for cows in the upper two quartiles (Figure 2B). Finally, previous studies have depicted increased occurrence of ovulation failure observed during the warmer months of the year (Thatcher and Collier, 1986; López-Gatius et al., 2005). In line with these observations, Primo et al. (2026) noted that the benefit of treating cows with GnRH at AI was observed during periods with increased temperature-humidity index (**THI**). Marginal predictions of P/AI at THI representing the average of quartiles 3 and 4 for THI was greater for cows treated with GnRH compared with untreated controls. On the other hand, P/AI for cows inseminated at THI representing the average of quartiles 1 and 2 revealed no benefit from treatment on P/AI (Figure 2C).

## Early identification of nonpregnant cows

Early identification and reinsemination of nonpregnant cows are paramount to maximizing pregnancy rates in dairy herds. Estrus detection is an effective strategy to reduce the interval to reinsemination (Giordano et al., 2012; Galvão et al., 2013). However, 30 to 45% of all nonpregnant cows are only identified at pregnancy diagnosis, even dairies with efficient estrus detection. Evaluation of luteal blood flow using Doppler ultrasonography allows for accurate identification of nonpregnant cows between days 20 and 24 after AI, with negative predictive values ranging from 98 to 100% following adequate methodology by experienced technicians (Madoz et al., 2022; Pugliesi et al., 2023; De Silva et al., 2024). Application of Doppler ultrasonography for identification of nonpregnant cows has a large potential to shorten reinsemination intervals in herds that rely solely or primarily on timed AI. On the other hand, the potential impact of nonpregnancy diagnosis based on CL perfusion in dairies that combine estrus detection and timed AI remains limited. Cows diagnosed as nonpregnant based on the absence of a vascularized CL between days 20 and 24 after AI largely coincide with cows that are detected in estrus and reinseminated within the same timeframe, which might represent 55 to 70% of all nonpregnant cows. In addition, false discovery rate ranges from 23 to 54% in lactating dairy cows (Madoz et al., 2022; Silva et al., 2024; Ferraz et al., 2024), retaining the need for a confirmatory evaluation based on the presence of a viable embryo one to two weeks later.

Interferon-tau (**IFNT**) is secreted into the uterine lumen by the elongating conceptus from 7 to 25 days of gestation (Farin et al., 1990). Studies focused on the expression of interferon-stimulated genes suggested that IFNT escapes from the uterus through the cervical canal (Kunii et al., 2018). Using a sandwich ELISA specific to bovine IFNT, researchers at Colorado State University demonstrated that the concentration of IFNT is greater in the cervical canal of pregnant cows compared with nonpregnant counterparts (Bishop et al., 2025). Furthermore, measurements of the concentration of IFNT in swab samples collected at the cervical canal or at the vaginal fornix guided by a digital vaginoscope further support the theory that IFNT leaks from the uterine lumen through the cervix (Newman et al., 2026). False negative rates ranged from 0 to 6% throughout the development of the assay and optimization of the device containing the cervical swab used to sample fluid from the cervical canal (Bishop et al., 2025). In a recent study (Bisinotto, unpublished data), false negative rates for identification of nonpregnant cows based on detection of IFNT in cervical swabs on day 16 or 17 after AI were 6 and 3%, respectively. Preliminary data on ovarian structures indicated that all cows diagnosed as nonpregnant on day 16 and 17 had CL ≥ 20 mm and follicles ≥ 10 mm. These results highlight that early nonpregnancy diagnosis based on cervical IFNT concentrations can be performed without relying on structural luteolysis. Moreover, the expected proportion of cows with functional CL (Bicalho et al., 2008) and follicles with ovulatory capacity (Sartori et al., 2001) support the development of strategies for synchronization of estrus or ovulation. Nevertheless, the economic impact of false negative results compared with potential gains from reducing time to reinsemination is yet to be calculated comprehensively.

## Conclusions

Fertility responses in lactating dairy cows have improved greatly in the past two decades. Besides major developments and wide implementation of timed AI programs and estrus detection technology, genetic selection for fertility traits and major strides in cow comfort, health, and nutrition have contributed to such increase in reproductive efficiency. Further optimization of reproductive management practices will continue to rely on the integration between the biological processes that control fertility and technological developments that can improve such processes.

## Data Availability

Research data is not available.
